# Reactive Oxygen Species (ROS): Beneficial Companions of Plants’ Developmental Processes

**DOI:** 10.3389/fpls.2016.01299

**Published:** 2016-09-27

**Authors:** Rachana Singh, Samiksha Singh, Parul Parihar, Rohit K. Mishra, Durgesh K. Tripathi, Vijay P. Singh, Devendra K. Chauhan, Sheo M. Prasad

**Affiliations:** ^1^Ranjan Plant Physiology and Biochemistry Laboratory, Department of Botany, University of AllahabadAllahabad, India; ^2^DD Pant Interdisciplinary Research Laboratory, Department of Botany, University of AllahabadAllahabad, India; ^3^Government Ramanuj Pratap Singhdev Post Graduate CollegeBaikunthpur, India

**Keywords:** reactive oxygen species signaling, plant growth and development, programmed cell death, seed germination, NADPH oxidases

## Abstract

Reactive oxygen species (ROS) are generated inevitably in the redox reactions of plants, including respiration and photosynthesis. In earlier studies, ROS were considered as toxic by-products of aerobic pathways of the metabolism. But in recent years, concept about ROS has changed because they also participate in developmental processes of plants by acting as signaling molecules. In plants, ROS regulate many developmental processes such as cell proliferation and differentiation, programmed cell death, seed germination, gravitropism, root hair growth and pollen tube development, senescence, etc. Despite much progress, a comprehensive update of advances in the understanding of the mechanisms evoked by ROS that mediate in cell proliferation and development are fragmentry and the matter of ROS perception and the signaling cascade remains open. Therefore, keeping in view the above facts, an attempt has been made in this article to summarize the recent findings regarding updates made in the regulatory action of ROS at various plant developmental stages, which are still not well-known.

## Introduction

**FIGURE 1:**
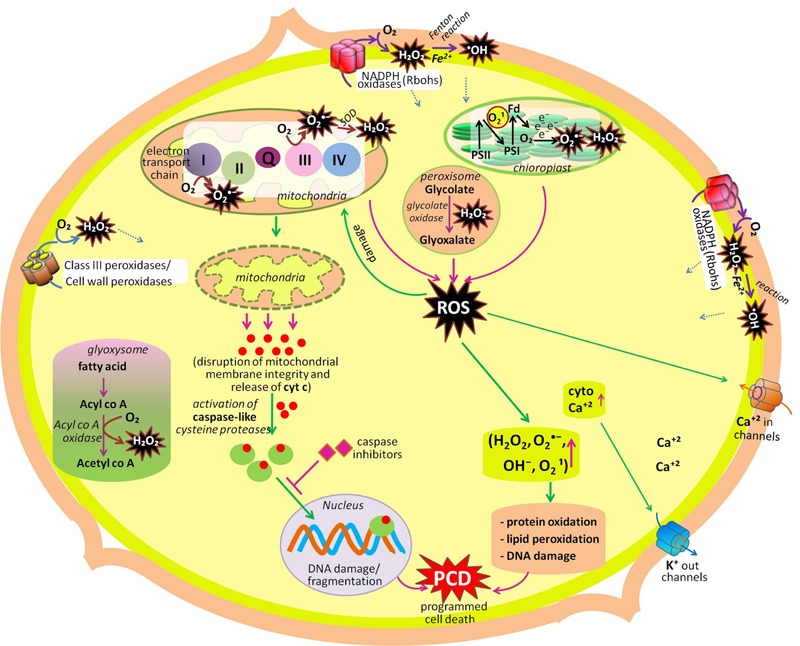
**Summary of production and metabolic fate of various ROS (hydrogen peroxide, superoxide radical, singlet oxygen, hydroxyl radical) in different cellular compartments (cell wall, chloroplast, mitochondria, peroxisome, glyoxysome, cytosol, plasma membrane).** Among these organelles, chloroplast, mitochondria, peroxisome, and plasma membrane actively participate in developmental programmed cell death (dPCD). Another organelles mentioned in the figure participates in other developmental process of the plant like seed germination, cell proliferation and differentiation, polar cell growth in root hairs and pollen tube, leaf development, etc. (modified after Gadjev et al., 2008; Mittler and Blumwald, 2010; Mittler et al., 2011; Petrov et al., 2015).

Life on the earth began under a reducing atmosphere. About ∼2.7 billion years ago, the introduction of O_2_-evolving photosynthetic organisms led to an accumulation of O_2_ that changed the reducing environment into an oxidized one. Since then, reactive oxygen species (ROS) have been unavoidable companions of aerobic life (Gill and Tuteja, 2010; Bhattacharjee, 2012). Electron transport systems (ETCs) generally produce ROS by virtue of O_2_ being a powerful electron acceptor. ROS such as superoxide radical ($O_{2}^{•–}$), hydroxyl radical (^•^OH), hydrogen peroxide (H_2_O_2_) and singlet oxygen (^1^O_2_) are either the product of oxidation–reduction (redox) reactions, or activated derivatives of O_2_, continually generated in chloroplasts, mitochondria, peroxisomes, and glyoxysomes (Sharma et al., 2012; Suzuki et al., 2012; Sandalio et al., 2013; Singh et al., 2015a,b), and also in the cytosol, apoplast, nucleus, and, endomembrane systems (Gechev et al., 2006; Ashtamker et al., 2007). These ROS are highly reactive and toxic, causing oxidative damage to macromolecules such as lipids, proteins, and nucleic acids (Karuppanapandian et al., 2011; Kapoor et al., 2015; Prasad et al., 2015). In photosynthetic tissues (leaves), chloroplasts are the major sources of ROS generation in plants (Asada, 2006; Dietz, 2016; Takagi et al., 2016; **Figure 1**; **Table 1**). Photosynthetic electron transfer chains of chloroplast produce high amounts of $O_{2}^{•–}$ (through leakage of electrons from the acceptor side of photosystem II to O_2_, from Fe–S centers of photosystem I and reduced ferredoxin (Mehler reaction; Pospisil, 2012), while in non-photosynthetic tissues of plants (i.e., roots, meristems, or seeds) mitochondria are the biggest sources of ROS generation (Navrot et al., 2007; Kalogeris et al., 2014; Huang et al., 2016). In mitochondria, the key sources of ROS production are NADH dehydrogenase complexes I and III, and the ubiquinone pool (Marchi et al., 2012; Steffens, 2014) where $O_{2}^{•–}$ radicals are generated from the complexes as a by product of energy metabolism by the reduction state of ubiquinone pool (Rhoads et al., 2006; Drose and Brandt, 2012). Other sources of ROS, mainly in non-photosynthetic tissues are NADPH oxidases, cell wall peroxidases, peroxisomes, and glyoxysomes. In glyoxysomes and peroxisomes, H_2_O_2_ is produced during fatty acid oxidation (by Acyl CoA oxidase), and photorespiration (by glycolate oxidase), respectively (del Rio et al., 2006; Gilroy et al., 2016; Kerchev et al., 2016; Rodríguez-Serrano et al., 2016). In chloroplasts, during impairment of CO_2_ fixation, an increased activity of ribulose-1,5-bisphosphate carboxylase/oxygenase leads to the formation of glycolate, which moves to peroxisomes and leads to the formation of H_2_O_2_, via its oxidation in the presence of glycolate oxidase enzyme (**Figure 1**; **Table 1**). Cell wall-associated extracellular peroxidases and plasma membrane-bound NADPH oxidases (e.g., *Nox, Rbohs* or respiratory burst oxidase homologs) are the key enzymes that produce H_2_O_2_ and $O_{2}^{•–}$ (usually rapidly dismutated to H_2_O_2_) in the apoplast (Bindschedler et al., 2006; Sagi and Fluhr, 2006; Choi et al., 2007). In *Arabidopsis*, 10 genes encoding respiratory burst oxidase homologs (Rbohs, i.e., *RbohA-RbohJ*) have been reported (Bedard and Krause, 2007; Marino et al., 2012). Notably, in presence of redox-active metals such as Fe^2+^ and Cu^+^, H_2_O_2_ will give rise to highly reactive ^•^OH (the most toxic oxidant in the ROS family) or $O_{2}^{•–}$ via the Fenton or Haber–Weiss reactions, respectively (Jomova et al., 2010).

**Table 1:** Various sites of ROS production and their role in growth and development of plants.

	Sources	Cellular localization	Developmental process	Reference
**ROS**
$O_{2}^{•–}$ and H_2_O_2_	Electron transport chain	Mitochondria	Seed germination	Moller, 2001; Noctor et al., 2007
$O_{2}^{•–}$ and H_2_O_2_	Fatty acid oxidation	Glyoxysome	Seed germination	Huang et al., 1983
H_2_O_2_	NADPH oxidase	Plasma membrane	Seed germination	Ishibashi et al., 2010
H_2_O_2_		Peroxisome	Seed germination	Palma et al., 2009
$O_{2}^{•–}$ and H_2_O_2_	NADPH oxidase	Plasma membrane	Root growth and root hair development	Foreman et al., 2003
H_2_O_2_ and $O_{2}^{•–}$	NADPH Oxidases	Plasma membrane	Pollen tubes growth	Cárdenas et al., 2006
H_2_O_2_	Class III peroxidases	Cell wall	Leaf development	Lu et al., 2014
**dPCD**				
$O_{2}^{•–}$ and H_2_O_2_	Electron transport Chain, NADPH oxidase	Mitochondria, Plasma membrane	Self-incompatibility in pollen	Wang and Zhang, 2011; Wang et al., 2013
$O_{2}^{•–}$ and H_2_O_2_	NADPH oxidase	Plasma membrane	Synergid cells dPCD	Duan et al., 2010, 2014
H_2_O_2_	NADPH oxidase	Plasma membrane	Tapetal dPCD	Xie et al., 2014
$O_{2}^{•–}$ and H_2_O_2_	NADPH oxidase	Plasma membrane	Formation of lysigenous aerenchyma	Rajhi et al., 2011
H_2_O_2_	NADPH oxidase	Plasma membrane	Secondary wall formation (lignification)	Potikha et al., 1999
H_2_O_2_	Electron transport chain	Chloroplast	Leaf senescence	Chen et al., 2012
H_2_O_2_		Peroxisome	Leaf senescence	Palma et al., 2009

Parallel to the production of ROS, aerobic organisms have evolved sophisticated and well-outfitted antioxidant defense machinery. This machinery possesses highly efficient enzymatic [superoxide dismutase (SOD); ascorbate peroxidase (APX); catalase (CAT); monodehydroascorbate reductase (MDHAR); dehydroascorbate reductase (DHAR); glutathione reductase (GR); glutathione peroxidase (GPX); glutathione-*S*- transferase (GST); and guaiacol peroxidase (GOPX)] and non-enzymatic [ascorbic acid (ASA); glutathione (GSH); tocopherols, phenolic compounds, and non-protein amino acids] antioxidant defense systems to control over the cascades of uncontrolled oxidation (Gill and Tuteja, 2010) to detoxify ROS in order to balance the cellular ROS level, as the maintenance of redox homeostasis is essential (Singh et al., 2012a,b; Wrzaczek et al., 2013). However, disturbance in the equilibrium between ROS and antioxidant defense system creates a condition of oxidative stress.

Although early research was focused on the toxic nature of ROS, the interest has shifted over the last decade toward their emerging role as signaling molecules in a broad range of physiological processes, such as growth and development, seed germination, programmed cell death (PCD), root growth, and gravitropism (Mittler et al., 2011; Wrzaczek et al., 2013; Baxter et al., 2014). A tight balance between ROS production and scavenging is necessary for the regulatory action of ROS (de Pinto et al., 2012; Sharma et al., 2012; Baxter et al., 2014). The uses of ROS as signaling molecules indicate that plants have evolved the ability to achieve a high degree of control over ROS toxicity (Bhattacharjee, 2012; Mattila et al., 2015). In this review, we have summarized studies from past decade that have improved our understanding about roles of ROS in signaling and the regulation of cellular processes in relation to plant growth and development.

## Cellular Redox and Signal Transduction

Coupled oxidation–reduction (redox) reactions in cells are the necessity of life. Various cellular signaling events are mainly based on redox reactions; therefore, it is probable that ROS are directly linked to the cellular redox metabolism. A redox regulatory network is found in each cell whose state is adjusted by ROS and these ROS regulate gene expression, translation, metabolism, and turnover (Dietz, 2016). Accordingly, cell maintains the redox homeostasis by powerful and complex antioxidants such as ascorbate and glutathione and/or antioxidant enzyme systems. Ascorbate and glutathione are much more than simple antioxidants, as they consist of both oxidizing and reducing forms. The antioxidants are principally maintained in the reduced state. A powerful reductant not only favors ROS removal but can also promote ROS generation (Foyer and Noctor, 2016). Alterations in the equilibrium of reduced *vs*. oxidized forms of the antioxidants might be used as a sensor for changes in the environment, and changes in ROS levels which might affect the redox status of the cell. Increased levels of ROS may result into the oxidation of antioxidant systems which change the redox equilibrium of the cell. According to Kleine and Leister (2016) in order to allow appropriate retrograde signaling to the nucleus, coordination of gene expression in between the compartments of the cell needs monitoring of chloroplast, glyoxysome, mitochondrial and peroxisome status. These facts imply that cells have evolved strategies to operate ROS as biological signals that control various developmental programs (Mattila et al., 2015). This explanation is based on the statement that a ROS can interact with a specific target molecule which perceives the elevated ROS concentration, and after that translates this information into a change of gene expression. These changes in transcriptional activity might be achieved by the oxidation of components of signaling pathways which consequently activate transcription factors (TFs) or directly by modifying a redox-sensitive TF. ROS effects on components of the mitogen activated protein kinases (MAPKs) cascade result in the indirect activation of TFs. In *Arabidopsis*, H_2_O_2_ activates *Arabidopsis thaliana* MPK6 (AtMPK6) and AtMAPK3 by the activity of MAPKKK *Arabidopsis* NPK1-RELATED PROTEIN KINASE1 (ANP1; Apel and Hirt, 2004) and strongly induces *A. thaliana* NUCLEOTIDE DIPHOSPHATE KINASE2 (AtNDPK2; Banfi et al., 2004). In a recent study, a link between the ROS-response ZAT12 zinc finger protein and iron regulation in cells was explored which suggests that the equilibrium between ROS and iron is crucial for the growth and development of plants preventing the formation of highly toxic ^•^OH (Le et al., 2016). According to Le et al. (2016) ZAT12 interacts and suppresses the function of FER-LIKE IRON DEFICIENCY-INDUCED TRANSCRIPTIONFACTOR, a central regulator of iron deficiency responses. In response to elevated ROS, ZAT12 is up-regulated and suppresses iron uptake thereby preventing the risk of ^•^OH formation. Another example of biological regulatory circuit was reported by Adachi et al. (2015) who have identified a WRKY TF that is phosphorylated by MAPK and a W-box in the promoter region of *Nicotiana tabacum* RBOH, interconnecting the phosphorylation events of MAPK in response to pathogen recognition with the accumulation of RBOH protein. Moreover, in recent years different members of the NAC family of TFs (e.g., Fang et al., 2015; Chen et al., 2016; Zhu et al., 2016), APETALA2/ethylene response TF REDOX RESPONSIVE TRANSCRIPTION FACTOR1 that are regulated by different WRKYs (Matsuo et al., 2015), and different zinc-finger proteins such OXIDATIVE STRESS2 (He et al., 2016) like ROS-response regulatory proteins were identified. Besides TFs, calcium (Ca) waves comprise important components of systemic signaling in plants (e.g., Suzuki et al., 2013; Carmody et al., 2016; Choi et al., 2016; Evans et al., 2016). Several findings summarized that RBOH acts as a central hub in the cellular ROS-signaling network (Baxter et al., 2014; Willems et al., 2016) and this RBOH functions along with MAPK pathways to integrate ROS signals and modulate cell-to-cell signal propagation in local and systemic signaling (Evans et al., 2016; Gilroy et al., 2016). These MAPK pathways take part in retrograde signaling from the chloroplast to the nucleus (Vogel et al., 2014; Dietz, 2016). Thus, MAPK pathways are of fundamental as well as far-reaching importance in converting ROS signals into protein phosphorylation.

## Role of ROS in Plant Growth and Development

### ROS and Cell Proliferation and Differentiation: A Cascade of Signaling Network

**FIGURE 2:**
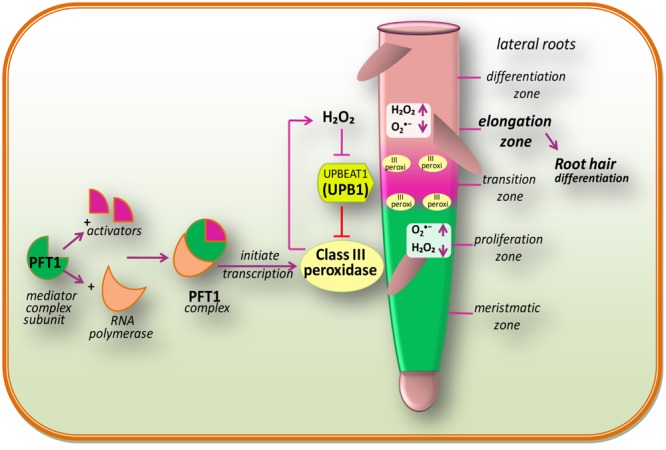
**Redox regulation during cell proliferation and differentiation and also a proposed model of PHYTOCHROME AND FLOWERING TIME1 (PFT1)-regulated root hair differentiation (modified after Sundaravelpandian et al., 2013).** The PFT1 mediator complex subunit interacts with their activators and RNA polymerase II to initiate the transcription. The PFT1 upregulates class III peroxidases which result into the production of H_2_O_2_ in the elongation zone while $O_{2}^{•–}$ is produced by NADPH oxidases in the meristmatic zone. These cellular processes overlap within the “transition zone.” A gradient of $O_{2}^{•–}$ and H_2_O_2_ in the root controls the transition between cell proliferation and differentiation. The daughter stem cell divides in the proliferation zone, which is characterized by high $O_{2}^{•–}$ level. Once after reaching into the transition zone, they come across an increased H_2_O_2_ level and stop dividing and initiate to elongate and differentiate in the differentiation zone. The bHLH transcriptional factor, UPB1, regulates meristem size thereby balancing the $O_{2}^{•–}$/H_2_O_2_ ratio by modulating the expression of PEROXIDASE genes (modified after Tsukagoshi et al., 2010).

In multicellular organisms, growth mainly depends on the maintenance of an appropriate equilibrium between cell division and differentiation (**Figure 2**). In the case of animals, interruption of this equilibrium between cell division and differentiation can lead to tumoral growth and disease, while in plants; it can lead to premature cessation of organogenesis, or as a consequence of abnormal growth (Zhang et al., 2008). The initial stage of differentiation is marked by the transition from cellular proliferation to elongation, which is regulated by ROS homeostasis (Tsukagoshi et al., 2010). According to Dunand et al. (2007), $O_{2}^{•–}$ and H_2_O_2_ are two main ROS, which are differentially distributed within the root tissues of model plant *Arabidopsis*. $O_{2}^{•–}$ principally accumulates in expanding meristem cells, while H_2_O_2_ accumulates in the elongation zone (Wells et al., 2010) and an overlap of both types of ROS is observed within the “transition zone” (**Figure 2**). The transition between root cell proliferation and differentiation is mainly controlled by the delicate equilibrium between $O_{2}^{•–}$ and H_2_O_2_, which in turn is regulated by a TF: UPBEAT1 (UPB1; Tsukagoshi et al., 2010). The UPB1, a member of the basic/helix loop-helix (bHLH) TF family, shows an increased expression in the root transition zone (Tsukagoshi et al., 2010). Tsukagoshi et al. (2010) have reported that plants over-expressing UPB1 had shorter roots due to a decrease in both meristem size and mature cells, while plants lacking UPB1 (*upb1* mutant) had longer roots with increased meristem size and longer root cells. Furthermore, when Tsukagoshi et al. (2010) investigated the mechanism of action of UPB1 regulated genes, a set of peroxidases (Prxs) was seen to be directly repressed by UPB1. Further investigation pointed out that $O_{2}^{•–}$ production was reduced in lines over-expressing UPB1 but increased in the elongation zone in the *upb1* mutant, while H_2_O_2_ was found to be increased in the plants over-expressing UPB1 and reduced in the elongation zone of *upb1* mutant. These findings suggested that the position of the transition zone is determined by the ramp formed by $O_{2}^{•–}$ in the meristem to maintain the cellular proliferation, and H_2_O_2_ in the elongation zone, for differentiation. This indicates that UPB1, feedback of which is regulated by H_2_O_2_, plays a key role in maintaining the balance of $O_{2}^{•–}$ and H_2_O_2_ via its control on Prxs expression.

Genetic analysis by Sundaravelpandian et al. (2013) signifies that the subunits of the mediator complex PHYTOCHROME AND FLOWERING TIME1 (PFT1)/MED25 and MED8 are vital for the differentiation of root hairs (**Figure 2**). Moreover, studies have demonstrated that PFT1/MED25 restricts cell growth, while MED8 works independently of PFT1 to control organ growth and regulates ROS balance in roots (Xu and Li, 2011). Transcriptional profiling of roots of *pft1* mutants disclosed that PFT1 triggers H_2_O_2_ formation mediated by a subset of class III peroxidase enzymes, to maintain the equilibrium between H_2_O_2_ and $O_{2}^{•–}$ during root hair differentiation. Perturbed H_2_O_2_ and $O_{2}^{•–}$ distribution was seen in *pft1* mutants, suggesting that PFT1 is necessary to maintain the redox homeostasis in roots (Sundaravelpandian et al., 2013).

In the fungus *Neurospora crassa*, at the start of each cell differentiation step, a hyperoxidant state occurs that leads to the development of conidia (Gessler et al., 2007). According to Aguirre et al. (2005), the hyperoxidant state is transient and unstable, and occurs as ROS production exceeds the antioxidant capacity of the cell. It is well-known from this example that antioxidants inhibit developmental processes, which leads to the idea that ROS may be required for these processes in plants as well. In contrast, it is predictable that the deficiency of antioxidant enzymes will result in to elevated ROS levels, and improved cell differentiation will take place.

### ROS and Programmed Cell Death (PCD): An Approach of Life for Plants

Programmed cell death, which is critical in plant organogenesis and survival, is an integral cellular program employed by plants by which targeted cells terminate to demise under certain developmental (vegetative and reproductive) and stress conditions (Aken and Van Breusegem, 2015; Ambastha et al., 2015; Durme and Nowack, 2016). It is essential for removing unwanted diseases or damaged cells, controlling cell number and maintaining the homeostatic balance and thereby improving the endurance of organisms (Petrov et al., 2015; Singh et al., 2015).

Plant PCD is related with a number of developmental processes including embryo formation, degeneration of the aleurone layer in germinating seeds, formation of root aerenchyma and epidermal trichomes, differentiation of tracheary elements, tapetum degeneration, pollen self-incompatibility, floral organ abscission, leaf shape remodeling, and leaf senescence (Gechev et al., 2006). ROS such as H_2_O_2_ is identified as key modulators of PCD along with other biological processes such as growth and development (Gechev et al., 2006). Concerning developmental programmed cell death (dPCD), a division can be made between (1) differentiation induced PCD that occurs as final differentiation step in specific cell types, for instance, tracheary elements in xylem, the trichome/ root cap, or tapetum layer of anther (Plackett et al., 2011; Bollhöner et al., 2012; Fendrych et al., 2014), and (2) age-induced PCD, i.e., cells must die to form organs for appropriate functions or shapes (unisexual reproductive organs in dicots, aerenchyma tissue, leaf shape), or cells die as they accomplished their function and/or are no longer required (nectaries and petals of flowers after pollination, leaf senescence; Thomas, 2013). Nevertheless, some types of PCDs, including endosperm cell death or pollen self incompatibility (SI) does not fall into either of above proposed classes (van Doorn et al., 2011).

The life cycle of plant alternates between diploid sporophyte and haploid gametophyte phases. The sporophytic phase starts with zygote (2n) formation and ends with flower bearing adult plant while gametophytic phase starts with sporophytic meiosis and develops into male (pollen grain) and female (ovule) reproductive structures. In dPCD, plants thereby removing tissue or organs which are no longer necessary conserve its energy along with blocking the possible entry sites for pathogens.

According to Tripathi and Tuteja (2007), to protect the initial growth of the ovule, sepals, and petals after accomplishing their role may undergo dPCD, where ROS specifically H_2_O_2_ is known to be involved in the death of petal cells (Tripathi and Tuteja, 2007). ROS is produced from H_2_O_2_ therefore, the enzymes regulating H_2_O_2_ level showed differential expression in dPCD during senescence (Halliwell and Gutteridge, 1989). Panavas and Rubinstein (1998) have also reported an increase in ROS due to increased SOD and decreased CAT activities in daylily plant that results into dPCD.

In megaspore, the chalazal cells differentiated into the three antipodal cells and micropylar cells develop into one egg and the two synergid cells and in center of embryo two polar nuclei are present (Yadegari and Drews, 2004). The synergid cells produce signals which attract and guide the pollen tube growth, ultimately deteriorate in a programmed manner (Punwani et al., 2007). In *A. thaliana*, FERONIA (FER) receptor kinase is responsible for the production of ROS by NADPH oxidase via ROP small GTPase in synergid cells (Duan et al., 2010, 2014) leading to dPCD. ROS by activating Ca^2+^-permeable channels alter the cell wall extensibility. In *A. thaliana*, ROS production (necessary for pollen tube growth) by the pollen-specific NADPH oxidases: RbohH and RbohJ is mediated by ANX, which maintains a tip-focused Ca^2+^ gradient (Boisson-Dernier et al., 2013; Kaya et al., 2014; Lassig et al., 2014). The excess Ca^2+^ influx might be the reason of ROS-induced pollen tube rupturation/PCD (Duan et al., 2014). Likewise, synergid-derived ZmES4, a defensin-like peptide by opening the K^+^ channel induced rapid pollen tube rupturation in maize (Amien et al., 2010). These findings revealed that ROS and other secreted proteins regulate cell wall alteration for the reception of pollen tube through the functions of FER in synergid cells and ANX in pollen tubes. On the opposite end of the ovule, three antipodal cells undergo dPCD immediately before fertilization in mature embryo sacs of *A. thaliana*. It is reported that lifespan of antipodal cell is regulated by central cell so; ROS accumulation specifically in the mitochondria of the central cell might act as a signal in dPCD of antipodal cell in a non-cell autonomous way (Hayashi et al., 2001).

During development of male sex organs, ROS signatures dictate the correct timing of tapetal dPCD because in order to release pollen, tapetum cells must die (Hautegem et al., 2015; Durme and Nowack, 2016). In tapetal dPCD, H_2_O_2_ acts as a key regulator. In *A. thaliana* RBOHE, a tissue-specific NADPH oxidase is the major H_2_O_2_ contributor supporting tapetal cell death (Xie et al., 2014). According to Xie et al. (2014) both overexpression and deficiency of RBOHE eliminated usual ROS accumulation leading to the male sterility.

ROS also play a signaling role during self-incompatibility- (SI; to prevent inbreeding by the rejection of incompatible pollen) induced pollen PCD in *Papaver*. According to Wilkins et al. (2011), the simultaneous scavenging of H_2_O_2_ and NO suppressed SI PCD (Wilkins et al., 2011). SI leads into signal transduction that involves increased ROS, Ca^2+^, NO, activation of MAPK, and protein phosphorylation as it is supported by large number of evidences that ROS play a key role in SI response that activates dPCD in self-compatible pollen (Bosch et al., 2010; Wilkins et al., 2011; Serrano et al., 2012; Jiang et al., 2014). In poppy, SI is associated with ROS induced activation of Ca^2+^ signaling cascade followed by a release of mitochondrial cytochrome c into the cytosol and caspase-3-like enzyme activity (Bosch and Franklin-Tong, 2007), and process is mediated by the activity of MAPK cascade signaling (Li et al., 2006; **Figure 1**). According to Wilkins et al. (2011, 2015) Ca^2+^ influx boosts H_2_O_2_ levels to SI-inducing levels that finally triggered a signaling network leading PCD thereby ensuring that fertilization is not achieved by incompatible pollen.

During pollination, when stigma is receptive (ready to receive pollen grains), accumulates high H_2_O_2_ levels, and that H_2_O_2_ levels decrease when stigma starts to support pollen development (Zafra et al., 2010; Serrano and Olmedilla, 2012; Serrano et al., 2012). After that, the signal exchanges in between the stigma and the pollen, that might regulate the production of ROS and reactive nitrogen species (RNS) in both tissues (Serrano et al., 2012) takes place. The H_2_O_2_ level in the stigma which was increased before pollination was found decreased after pollen arrival. Contrary to this, the $O_{2}^{•–}$ and NO levels were increased with attendant increase in peroxynitrite (ONOO^-^; Serrano et al., 2015). Treatment with ONOO^-^ scavengers decreases papillar cell death and also reduces the quantity of pollen grains deploying dPCD, suggesting that ROS mediated PCD signaling takes place during incompatible pollination in the olive (Serrano et al., 2015). After enterance in female gametophyte, pollen tube discharges its content (two male gametes). Out of two male gametes, one fertilizes the binuclear central cell to form a triploid endosperm and other migrates to the egg cell (Yadegari and Drews, 2004). The endosperm functions as a storage tissue for developing embryo which after sometimes undergo ROS induced PCD mediated by abscisic acid (ABA) and ethylene.

During cereal seed germination, gibberellic acid (GA) is produced by plant embryo that activates the aleurone cells to release an amylase enzyme, which sequentially hydrolyzes and mobilizes starch from the endosperm in the seeds thereby providing energy to the embryo. After completion of germination, these aleurone cells are abolished by PCD. According to Yadegari and Drews (2004), ROS and GA play vital roles in the control and implementation of aleurone PCD (discussed in next section).

Besides embryonic development, ROS also play a key role in dPCD of vegetative parts; the development of xylem tracheary elements is one of them. Contrary to other cell type in plants, xylem tracheary elements are functionalized by PCD leading into hollow cell cadavers with merely a remaining cell wall demarcating the sap conducting cylinder (Roberts and McCann, 2000). H_2_O_2_ by increasing cross-linking of polymers induces stiffening of the cell wall as growth ceases next to onset of differentiation (Hohl et al., 1995; Schopfer, 1996). Lignification in stem of *Zinnia elegans* L. is characterized by a burst in H_2_O_2_ production, which could act as a developmental signal in secondary wall differentiation (Potikha et al., 1999). This H_2_O_2_ may coordinate and regulate at the transcriptional level mRNAs synthesis encoding phenylalanine ammonia-lyase like lignin biosynthetic enzyme (Desikan et al., 1998), and xylem peroxidases (Wu et al., 1997) or trigger xylem differentiation thereby inducing PCD (Fukuda, 1996) and formation of secondary cell wall. The major H_2_O_2_ production site in the differentiating xylem is outer-face of the plasma membrane of both non-lignifying xylem parenchyma cells and differentiating thin-walled xylem cells. From their sites of production, H_2_O_2_ diffuses (mainly through the continuous cell wall space) to the differentiating (cells which are at the state of secondary cell wall-formation) as well as differentiated xylem vessels (cells already have completed secondary cell wall formation, i.e., lignified; Ros Barceló, 2005), and H_2_O_2_ is thought to not limit the rate of xylem lignification (Ros Barceló, 2005). These findings strongly supported that H_2_O_2_, which is a key component for inducing dPCD signaling network in lignifications of xylem vessels, is mainly produced from non-lignifying xylem parenchyma cells as it is necessary for the polymerization of cinnamyl alcohols.

A similar picture has been reported in various forms of developmental cell death— formation of aerenchyma and the senescence-associated cell death. According to del Rio et al. (2006), ROS like $O_{2}^{•–}$ and H_2_O_2_ are the key coordinators of senescence (Lam, 2004). In the internodes of rice, the exogenous application of H_2_O_2_ promotes the formation of lysigenous aerenchyma (Steffens et al., 2011). In cortical cells of maize roots, Rajhi et al. (2011) have reported a strong up-regulation of gene encoding RBOH, thereby suggesting that RBOH mediated ROS generation participates in the formation of lysigenous aerenchyma.

In many plants, trichomes in their fully differentiated stage are dead. The development of trichome trails a switch from mitosis to endoreduplication, branching of cells, expansion, and ultimate cell death initiated by H_2_O_2_ burst (Hulskamp, 2004). In trichomes of succinic semialdehyde dehydrogenase (key enzyme of γ-aminobutyrate metabolic pathway) knockout plants, enhanced H_2_O_2_ levels are observed (Bouche et al., 2003). In the stem nodes of rice plants, the epidermal cells that cover the primordia of adventitious roots undergo cell death before the emergence of adventitious root. Ethylene promotes epidermal cell death, which is mediated by an excess H_2_O_2_ production (Steffens and Sauter, 2009). In rice plant, a non-enzymatic H_2_O_2_ scavenger metallothionein *MT2b* when downregulates it promotes ROS level in cell (Wong et al., 2004). In case of epidermal cells, ethylene downregulates *MT2b* that results into PCD thereby revealing that ethylene promotes ROS accumulation that lead to *MT2b* mediated cell death (Steffens and Sauter, 2009).

Significant roles of ROS have also been reported during natural course of senescence, the last step of leaf development in the life span of an annual plant (Lee et al., 2012; Springer et al., 2015). The accumulation of ROS have been suggested to be an age-associated factor that triggers leaf senescence (Khanna-Chopra, 2012). Foyer and Noctor (2005) proposed that ROS-triggered senescence is not caused by physicochemical damage of the cell, but that these molecules (ROS formation due to leakage of electrons from ETC to O_2_ at the onset of senescence) behave as signals, which activate gene expression pathways leading to suicide events (Sandalio et al., 2013; Jajic et al., 2015). During cell senescence, lipid peroxidation can be activated either by lipoxygenases in some tissues, where the activity of lipoxygenase increases with increasing senescence, or by ROS (Chopra, 2012; Bhattacharjee, 2014). Therefore, it was concluded that, during senescence, lipoxygenase plays an essential role in promoting oxidative damage, in which not only stimulates lipid peroxidation but it can also form ^1^O_2_. Vanacker et al. (2006) have also reported that in senescent leaves, H_2_O_2_ content increases in parallel with an increase in lipid peroxidation and protein oxidation. So it is obvious that H_2_O_2_ plays a crucial role in the regulation of senescence signaling.

After senescence, the resulting plant organ detaches from main plant body and the process is called abscission. In several studies, it has been reported that Prxs are regularly expressed during diverse types of abscission (Meir et al., 2006; Cai and Lashbrook, 2008).

Before discussing the role of ROS in leaf abscission, the notion should be recall that Prxs actively participate in the cell wall loosening (Schweikert et al., 2000; Schopfer et al., 2001). Sakamoto et al. (2008) demonstrated that continuous H_2_O_2_ production in the cell wall participates in leaf abscission signaling, which is needed to induce the expression of cellulase enzyme (the cell wall-degrading), and when this continuous H_2_O_2_ production was suppressed by ROS inhibitors, it inhibited the cellulase activity and consequently inhibited abscission. On the other hand, exogenous application of H_2_O_2_ enhanced cellulase expression and abscission therefore, signifying that H_2_O_2_ production is essential to induce abscission. After continuous cellulase production (induced by H_2_O_2_), enhanced H_2_O_2_ generation at the Abscission-Zone in the Abscission-Zone-separating period was observed that might play a role in the cleavage of wall polysaccharides and loosen the cell wall (Gapper and Dolan, 2006). Therefore, a huge ROS production that probably driven by Prxs at the late stage of abscission may be associated with the cell wall degradation process.

### ROS in Seed Dormancy and Early Seed Germination: A Hub of Regulatory Networks

**FIGURE 3:**
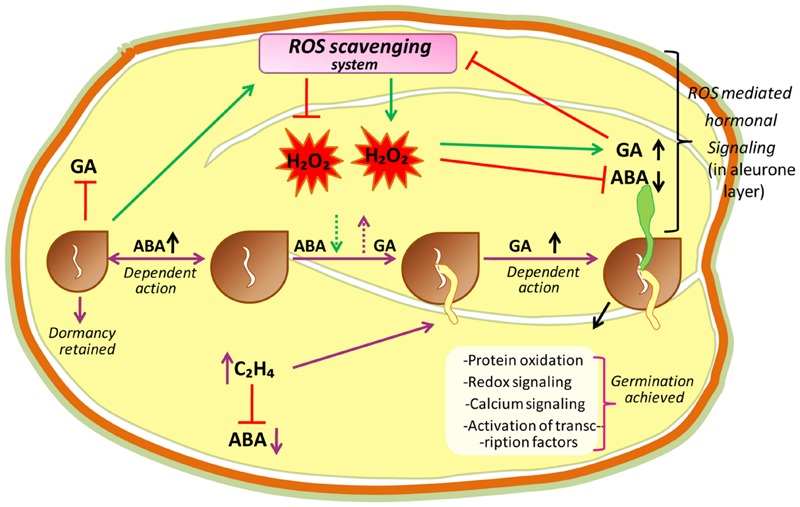
**A hypothetical model proposing central role of ROS in the mechanisms of seed dormancy release and germination.** The ROS core plays a central in the regulation of germination through the crosstalk with abscissic acid (ABA) and ethylene (C_2_H_4_). In dormant state, high amount of ABA maintains dormancy by inducing an active signaling pathway. In non-dormant seeds, ABA concentration lowered which is associated with higher ROS level. Apparently H_2_O_2_, which could in turn interfere with ABA and GA signaling pathways, modify redox status and induce protein oxidation, redox signaling, calcium signaling and transcriptional factor activation leading to the germination of seeds.

Seed dormancy and seed germination mechanisms are essential processes in plant development, and are believed to be a part of a complex regulatory network (**Figure 3**). In this network, ROS are considered to be key signaling actors as reported in *A. thaliana* (Liu et al., 2010; Leymarie et al., 2012), barley (Ishibashi et al., 2010; Bahin et al., 2011), cress (Müller et al., 2009), wheat (Ishibashi et al., 2008), and sunflower (Oracz et al., 2007). Seed germination starts with water uptake, along with initiation of cell division, and ends with radicle protrusion (Holdsworth et al., 2008; Bewley et al., 2013). In dry seeds, due to highly reduced enzyme activities, ROS are probably originated from lipid peroxidation like non-enzymatic reactions that occur even at very low moisture contents (Vertucci and Farrant, 1995; McDonald, 1999). However, in hydrated seeds (that occurs following imbibition) ROS may generate from all metabolically active compartments like chloroplasts (by election transfer in photosystems), glyoxysomes (by lipid catabolism), mitochondria (via respiratory activity), peroxisomes (by purine catabolism), and plasma membranes (by NADPH oxidase; Bailly, 2004). In hydrated seeds, the continuation of respiration (in mitochondria) in imbibed seeds can lead to electron leakage because the electrons of mitochondrial electron-transfer chain (mETC) have sufficient free energy to directly reduce the O_2_, and this can be considered as unavoidable source of increased ROS production in mitochondria (Rhoads et al., 2006; El-Maarouf-Bouteau and Bailly, 2008). Due to this reason, in hydrated seeds, the mitochondrial activity is considered as major source of ROS (such as H_2_O_2_) production during germination (Noctor et al., 2007). Moreover, in hydrated seeds, ROS targets may be close or far from their production sites as the free water allows ROS (or their longer-living or movable forms like H_2_O_2_) to move and reach their targets farther from their production sites while, in dry seeds the ROS targets must be close to their production sites (Bailly et al., 2008). It has been reported that H_2_O_2_ is produced in the seeds of *Zinnia elegans* (Ogawa and Iwabuchi, 2001), maize (Hite et al., 1999), wheat (Caliskan and Cuming, 1998) and soybean (Puntarulo et al., 1988) during the early imbibitional period, and cellular responses to H_2_O_2_, which is the most labile ROS messenger, mostly rely on the involvement of redox-active proteins (Foyer and Noctor, 2013), stimulation of redox-sensitive TFs that orchestrate downstream cascades (Petrov and Van Breusegem, 2012), oxidation of specific peptides (Moller and Sweetlove, 2010), and the activation of MAPKs (Barba-Espín et al., 2011). ROS produced during imbibition in different sub-cellular compartments, largely affect the expressions of various genes (Neill et al., 2002). The sub-cellular ROS interact with specific target molecules (particular for increased ROS concentration), which there translate that information by altering the gene expression (Laloi et al., 2004). Oxidation of certain components that keep on to activate TFs of the signaling pathway, or the direct modification of redox-sensitive TFs by ROS, may led to the alteration of transcriptional activity (Laloi et al., 2004).

It has been reported in several studies that during seed imbibition, ROS oxidize specific proteins for driving the germination process (Barba-Espín et al., 2011; El-Maarouf-Bouteau et al., 2013), or oxidize mRNAs to prevent their translation (Bazin et al., 2011).

According to El-Maarouf-Bouteau and Bailly (2008), ROS have well-recognized role in endosperm weakening, protection against pathogens, mobilization of seed reserves, and PCD in seeds. In some seeds, an endosperm covers the radical tip and acts as a mechanical barrier in radicle protrusion.

When addressing the event of seed germination and seedling establishment, it appears exceedingly relevant to consider and investigate the possible interaction between ROS and plant hormones in the process of germination, as ROS involving PCD process have been established in aleurone layer of cereal grains, which are closely related to H_2_O_2_ interactions with GA and ABA hormones (Ishibashi et al., 2013; Corbineau et al., 2014; Miransari and Smith, 2014; **Figure 3**). It is known that in aleurone cells of cereals, GA supports germination as well as post-germination processes, which are inhibited by ABA. Although GA downregulates the ROS scavenging enzyme activities, they are retained by ABA, which advocates a ROS link to PCD execution. *In vivo*, ROS can damage/oxidize/weaken the cell wall polysaccharides, activate calcium channels, Ca^2+^ binding proteins, MAPK cascades, and damaged cell wall polysaccharides, permit elongation of the growing radicle, which is a characteristic feature of endosperm rupturation in the aleurone layer (Müller et al., 2009; Xu et al., 2010; Barba-Espín et al., 2011; Diaz-Vivancos et al., 2013).

These authors showed that wall polysaccharides are oxidized *in vivo* by the developmentally regulated action of apoplastic ^•^OH. The cell wall loosening is assumed to involve the enzymatic as well as non-enzymatic cleavage of structural polysaccharides (Passardi et al., 2004). The regulated action of apoplastic ^•^OH radical, which is generated by Prxs and/or the Fenton reaction in the cell wall (reaction 1) of radicles and endosperm caps, is known to cause the cleavage (breakdown) of hyaluronate (Stern et al., 2007), chitosan (Tanioka et al., 1996) and pullulan-like polysaccharides (Crescenzi et al., 1997).

(1) $${\text{Fe}}^{\text{2}}{}^{\text{+}}{\text{+H}}_{\text{2}}{\text{O}}_{\text{2}}\;⟶\;{\text{Fe}}^{\text{3}}{}^{\text{+}}{\text{+}}^{\cdot }{\text{OH+OH}}^{-}$$

The production as well as the action of ^•^OH increased during endosperm weakening and radicle elongation, and were inhibited by ABA. These outcomes were reversed by GA, representing a positive role of ^•^OH in cell wall loosening during seed germination (Müller et al., 2009).

When aleurone cells are exposed to GA, α-amylase and other hydrolytic enzymes are synthesized and secreted. Contrary to this, antioxidants (activities of CAT, APX, and SOD) suppressed the induction of amylases. While ABA acts as an antagonistic factor blocking the GA response in aleurone cells via the expression of a different set of genes- reinforcing the idea that H_2_O_2_ functions in GA and ABA signaling thereby regulating α-amylase production in aleurone cells (Nonogaki, 2014).

In seeds, a majority of studies have reported that ABA suppresses ROS generation, i.e., it acts as a negative regulator during seed germination (Ishibashi et al., 2012; Ye et al., 2012) while behaves as a positive regulator of the induction of dormancy (Finkelstein et al., 2008). In presence of GA, exogenous H_2_O_2_ had minor effect on the degradation of primary transcriptional repressor, i.e., DELLA proteins Slender1 (SLN1) mediating GA signaling, but it supported the production of the mRNA encoding GAMyb (GA-regulated Myb TF) that acts in downstream of SLN1 protein and engages α-amylase mRNA induction. Moreover, H_2_O_2_ holdbacks the production of ABA-responsive protein kinase (PKABA) mRNA, which is induced by ABA; the production of GAMyb mRNA is repressed by PKABA (Ishibashi et al., 2012). From these studies, Ishibashi et al. (2012) have concluded that the repression of GAMyb mRNA was released by H_2_O_2_ via PKABA and this H_2_O_2_ subsequently promoted the production of α-amylase mRNA, thereby suggesting that GA generated H_2_O_2_, act as a signal that antagonizes ABA signaling in aleurone cells.

Ethylene is another hormone that acts as a positive regulator of seed germination. In soybean seeds, Ishibashi et al. (2013) observed that during seed imbibition ROS are produced, which further promotes ethylene production in sunflower seeds. Recently, Arc et al. (2013) have reported that the interaction between ABA and ethylene have antagonistic effects during seed germination. Moreover, by giving special consideration to cross-talk between ABA, ethylene, and ROS, mechanisms of action of ROS in the process of germination have been elucidated (**Figure 3**).

According to Barba-Espín et al. (2011) ROS also participate in breaking seed dormancy and in germination by activating the oxidative pentose phosphate pathway (oxPPP). The authors observed that the exogenous application of H_2_O_2_ induces the carbonylation of enzymes taking part in restoration of reducing power during glycolysis, which stimulated oxPPP. Moreover, a relationship between H_2_O_2_ contents and gene expression related to MAPKs has also been noticed by Barba-Espín et al. (2011). According to Xu et al. (2010) during seed germination, MAPK cascades affect cell division and the hormone so, Barba-Espín et al. (2011) pointed out that variations in H_2_O_2_ contents (increases during germination) of seed may induce germination thereby activating those MAPK cascades.

### ROS and Root Gravitropism

After seed germination, the part of seedling which have to make root and shoot, sense the direction of the gravity vector and elicit gravitropic growth, generally sending the roots to downward and shoot to return to upward and directed growth. Generally it is accepted that growth responses reveal differential auxin redistribution to the flanks of the organ that results into reduction of elongation on one side and acceleration on the other. This differential growth in response to gravity leads to the bending of organ. These gravitropism responses have been connected to ROS generation in the roots of *Arabidopsis* and maize (Joo et al., 2001, 2005). In gravistimulated roots, ROS accumulation was found asymmetrical to the lower cortex within 30 min of reorientation, which becomes symmetrical upon longer stimulation (Joo et al., 2001). Asymmetrical application of H_2_O_2_ impregnated agar block causes root bending toward block. Here, some caution must be exercised such as H_2_O_2_ application will also interact with the wall that might have profound effect on cell extensibility. ROS are well-known to play a critical role in cell integrity that might also potentially lead to growth arrest. However, treatment with *N*-acetyl cysteine (NAC)-like ROS scavengers was observed to inhibit the response or curvature without affecting growth rate thereby suggesting that asymmetrical ROS generation is responsible for the gravitropic response (Joo et al., 2001). As the gravitropic responses are known to originate from inhibition of root elongation on the lower flank of the root, these findings are reliable with ROS accumulation to the lower flank acting as a growth inhibitor.

It was thought that auxin might be a primary regulator of such kind of root elongation where it is accumulated on the lower flank to inhibit the growth of gravistimulated root. Consistent with these thoughts, Schopfer et al. (2002) reported that auxin can evoke ROS generation in a process which shows a close parallels to the animal model of ROS generation via NAPDH oxidases (Joo et al., 2005). A similar story is also emerging of a potential role of ROS in the gravitropic response of the maize roots. The role of ROS in auxin-induced root gravitropic responses has been reported in *Zea mays* (Joo et al., 2001), and results strongly suggest that ROS-mediated auxin redistribution by gravity stimulates an increase in gravitropic curving. Firstly, Joo et al. (2001) noticed that ROS are formed subsequent to gravitropic stimulus. When maize roots were kept horizontally, the ROS generation was detected in the apex, stimulating the gravitropic response (Joo et al., 2001). Secondly, ROS production is increased by auxin application to root cells in intact plants as well as in root protoplast cultures (Cervantes, 2001). Further treatment of roots with *N*-1-naphthylphthalamic acid (NPA, an auxin transport inhibitor) results in inhibition of gravitropism and the effect of NPA can be reversed by adding H_2_O_2_ which has indicated a direct causal relationship between auxin, ROS, and gravitropism (Cervantes, 2001). It has been observed that root bending, after pretreatment with the auxin transport inhibitor NPA, was brought about by unilateral ROS application to vertical roots. However, ROS scavenging by the antioxidants NAC, trolox and ASA inhibits root gravitropism (Joo et al., 2001). However, the effect of NPA was reversed by addition of H_2_O_2_. Joo et al. (2001) pointed out that ROS might work by kinase activation, but also considered that other mediators of the gravitropic response, such as inositol (1,4,5)-trisphosphate and calcium, might be involved. Furthermore, Joo et al. (2005) observed that phosphatidylinositol 3-kinase is involved in auxin-mediated ROS production that regulates root gravitropism while retreatment with the phosphatidylinositol 3-kinase activity inhibitor LY294002 blocked the auxin- mediated ROS generation, decreasing the sensitivity of root tissue to gravistimulation (Joo et al., 2005).

It is well-known that in plant gravitropism, redistribution of auxin plays an important role (Petrasek and Friml, 2009) in ROS mediated root gravitropism. To find out the role of auxin and ROS, Lupini et al. (2013) used the auxin transport inhibitors 2,3,5-triiodobenzoic acid (TIBA) and naphthylphthalamic acid (NPA) and a secondary metabolite coumarin which are known to alter the gravitropic responses in the roots of *A. thaliana*. Coumarin itself did not show any change in gravitropic response, but when added with NPA or TIBA, it reversed the effect of inhibitors (Lupini et al., 2013). Moreover, Lupini et al. (2013) studied ROS distribution in root tips and reported that NPA or TIBA causes distribution of $O_{2}^{•–}$ around the root tip, which disappeared after coumarin addition to both treatments, restoring ROS localized distribution. These findings suggest that coumarin effect in restoring the root curvature did not depend upon auxin redistribution, but was mediated by ROS generation. Further to investigate the localization of ROS, Libik-Konieczny et al. (2015) by using blue formazan (NBT; precipitates $O_{2}^{•–}$ accumulation) and 3,3′-diaminobenzidine (DAB; for H_2_O_2_ accumulation) demonstrated that $O_{2}^{•–}$ was localized within the tip of root primordia, vascular cylinder cells as well as in the distal and middle parts of newly formed organs during the early stages of rhizogenesis; while H_2_O_2_ was pronounced in cortical and vascular bundle cells. On adding DPI to the medium, $O_{2}^{•–}$ accumulation was then restricted to epidermis cells, while that of H_2_O_2_ was limited in vascular tissues (Libik-Konieczny et al., 2015). These findings suggest that $O_{2}^{•–}$ engages itself in the process of rhizogenesis, while H_2_O_2_ is engaged in developmental processes such as cell growth.

### ROS in Growth and Development of Root Hair and Pollen Tube

In plant system, the tip growth in either of the cases is mainly associated with the deposition of membrane and new wall materials centered toward the apex of elongating cells, thereby resulting into the formation of tube-like structure. To produce a correct shape and size, cell expansion needs to be carefully regulated. Polar cell growth is continued by oscillatory feedback loops including three main components, i.e., ROS, Ca^2+^ and pH that together play an important role in regulating this process over time (Mangano et al., 2016). Apoplastic ROS balance controlled by NADPH oxidases and class III peroxidases has a great impact on cell wall properties during cell expansion (Mangano et al., 2016).

During expansion of polar cell (root or pollen), the apical zone is characterized by a tip-focused elevated cytoplasmic Ca^2+^ (_cyt_Ca^2+^) gradient and related proteins along with the apoplastic ROS (_apo_ROS) generation (Konrad et al., 2011; Qin and Yang, 2011; Hepler et al., 2012; Steinhorst and Kudla, 2013). Cell takes Ca^2+^ either from external sources or stored in the cell wall and this Ca^2+^ released by changing _apo_pH which is mainly controlled by plasma membrane located activation/deactivation of H^+^ pumps (AHA). Additionally, Ca^2+^ can be stored in vacuoles and ER-Golgi and released into the cytosol. To maintain low concentrations of _cyt_Ca^2+^, autoinhibitory P-type IIB Ca^2+^-ATPases (ACAs) transport Ca^2+^ back to the apoplast. In addition to this, the H^+^/Ca^2+^ antiporter activity of calcium-exchanger (CAX) translocates Ca^2+^ back to the apoplast and, at the same time, imports H^+^ into the cytoplasm. In the tip zone, high levels/concentration of _cyt_Ca^2+^ activates _apo_ROS generation by NADPH oxidases (NOXs). Moreover, high levels of ROS quickly elevate the concentration of _cyt_Ca^2+^ (Foreman et al., 2003; Duan et al., 2014) by an unknown phenomenon. NOXH/NOXJ (Wu et al., 2010; Boisson-Dernier et al., 2013) and NOXC (Foreman et al., 2003; Monshausen et al., 2007, 2008) were previously proposed to connect _apo_ROS production with the rapid activation of Ca^2+^ channels (CaCs) of plasma membrane in growing pollen tubes and root hairs, respectively. The idea that ROS are important for sustaining polar growth came into existence with the cloning of root hair defective 2 (*rhd2*) mutants of *A. thaliana* (Foreman et al., 2003). In *rhd2* mutant background, according to Monshausen et al. (2007) and Macpherson et al. (2008) root hairs correctly initiated their developmental program and form bulges on the epidermal cell side but they fail to elongate/transition to tip growth. When alleles of the *rhd2* were cloned, they were found to inhabiting a gene encoding the respiratory burst oxidase homolog C (AtRBOHC) of *Arabidopsis*, an NADPH oxidase similar to mammalian gp91phox (The phox91 family is conserved throughout the animal, plant and, fungal kingdoms; Foreman et al., 2003), responsible for ROS production during oxidative burst. It means that RHD2 plays an important role in electron transfer from NADPH to an electron acceptor, and resulting in ROS formation. Besides RHD2, another target of ROS signaling in growing root hair has been reported, i.e., OXIDATIVE BURST INDUCIBLE1 (OXI1) kinase (Rentel and Knight, 2004). OXI1 is a Ser-Thr kinase, which plays a role in elongation of root hairs, as it is slightly shorter in *oxi1* mutant than wild type. It was suggested that RHD2-mediated ROS leads to the activation of a MAPKs cascade through OXI1 during growth of root hairs.

A ROS burst is crucial for the rupturation of pollen tube and sperm release (Duan et al., 2014). Oscillatory growth is also related to changes in pH which is regulated by cation (H^+^)/anion (OH^-^)-permeable channels, by membrane H^+^-ATPases (AHA; Falhof et al., 2016) and antiporters (Ca^2+^/H^+^ exchangers). AHA is supposed to be responsible for pumping H^+^ out of pollen tubes remains to be identified while AHA2 is highly expressed in growing root hairs. AHA directly controls apoplastic pH (_apo_pH), which affects the enzymatic mechanism that alters cell wall components during cell expansion. Polar growth entails the stretching and deformation of the existing wall in the apical zone, which is escorted by the secretion of new wall materials (Altartouri and Geitmann, 2015). Oscillations in Ca^2+^, ROS and pH are coupled to transient cell wall loosening to facilitate turgor-driven localized cell expansion (Braidwood et al., 2014; Spartz et al., 2014; Wolf and Höfte, 2014). In cells, growth is determined by the balance between cell wall loosening and stiffening. Root hair growth has to occur not only in accordance with the loosening of the cell wall to allow the expansion at its tip, but also with a need to avoid tip bursting by maintenance of wall integrity. In order to attain this pattern, the factors engaged in pollen tube growth such as _cyt_Ca^2+^ levels, pH, and NADPH are found to oscillate in such a manner in which the highest concentration follows the highest peak in growth, in concert to maintain the periodic oscillations (Cárdenas et al., 2006; Lovy-Wheeler et al., 2006). In root hairs, the maximum of these oscillatory fluctuations in _apo_ROS concentration and apoplastic/cytoplasmic pH lead cell growth peaks by 7–8 s, while _cyt_Ca^2+^ oscillations delay oscillations in cell growth by approximately 5–6 s (Monshausen et al., 2007, 2008). In pollen tubes, the oscillations in _cyt_Ca^2+^ concentration are lagged by approximately 11 s in relation to cell expansion rates (Pierson et al., 1994). Thus, polar cell growth is improved, and perhaps rapidly repressed, by high concentration of _cyt_Ca^2+^ and, consequently, high _apo_ROS concentrations and a more alkaline _apo_pH.

In *Arabidopsis*, by using high-resolution growth measure-ments, Monshausen et al. (2008) showed that the frequency of growth pulses was approximately 3 per minute, similar to the frequency of pulses of ROS production and to the oscillations in the apical Ca^2+^ gradient. A comprehensive study of these oscillations implied a growth burst at 5 s due to an increase in tip-focused Ca^2+^ that may be mediated by a stretch-activated Ca^2+^ channel, which is followed 2 s later by ROS production in the wall (Monshausen et al., 2008). According to Monshausen and Gilroy (2009), this ROS production may play a role in covalent cross linking of polymers, and therefore strengthen the wall to prevent expansion and bursting. Monshausen et al. (2007) reported that tip growth ceased after the addition of external H_2_O_2_; however, scavenging of ROS promoted tip bursting, which suggests that ROS also play a role in wall rigidification. From these facts, it can be concluded that ROS-activated Ca^2+^-permeable channels induce Ca^2+^ influx to increase the _cyt_Ca^2+^ after the beginning of Rboh-mediated ROS generation, which further activates Rbohs. Therefore, Ca^2+^ increases in the cytoplasm probably response to increase ROS that alter Ca^2+^ influx.

**FIGURE 4:**
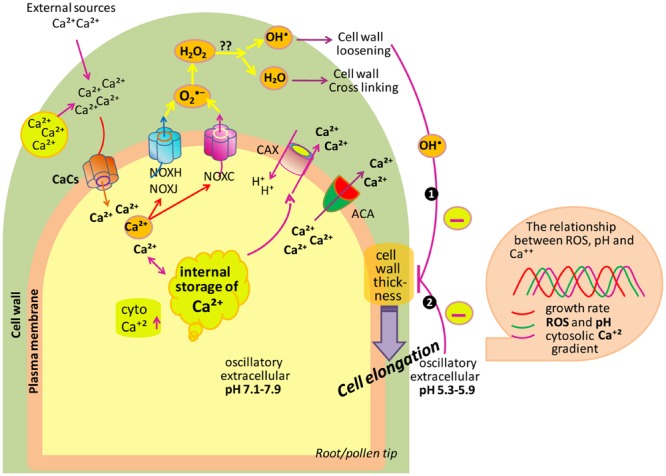
**Model depicting the role of ROS, Ca^2+^ and pH in tip growth of root/pollen cells.** Cell uptakes Ca^2+^ from its surrounding. To maintain the low levels of _cyt_Ca^2+^, ACAs transport Ca^2+^ back to the apoplast. Besides this, the H^+^/Ca^2+^ antiporter translocates Ca^2+^ back to the apoplast and, at the same time, imports H^+^ into the cytoplasm. The _cyt_Ca^2+^ activates NOXC and NOXH/NOXJ to produce _apo_ROS in root hairs and pollen tubes, respectively. NOX produces apoplastic $O_{2}^{•–}$, which is dismutated by SOD to H_2_O_2_. Also, _apo_H_2_O_2_ and O_2_ generate ^•^OH (in the hydroxylic cycle) which catalyzes the nonenzymatic cleavage of polysaccharides, thereby allowing tip growth. Box on right side showing that extracellular ROS, pH as well as _cyt_Ca^2+^ are coupled to growth oscillations. (Modified after Monshausen et al., 2009; Mangano et al., 2016).

If we are talking about ROS signaling, it is noteworthy that changes in extracellular pH correlate with the oscillations in growth, which permit the alteration of tip growth in root hairs of *Arabidopsis* (Monshausen et al., 2007). The stiffening of the cell wall is carried out by both ROS and increased pH, which make cell wall resistant to turgor pressure. In the growing tip, oscillation of Ca^2+^ gradients is followed by alkalinization peaks in the apoplast along with the constitutive $O_{2}^{•–}$ generation across the subapical part of the tip (Monshausen et al., 2009; Swanson and Gilroy, 2010). An oscillatory component of extracellular pH at the tip of pollen tubes has been reported by Messerli and Robinson (2007), that changes the phase by producing ROS and increases the growth by increasing the Ca^2+^ accumulation (Messerli and Robinson, 2007). A similar series of events ROS, pH, Ca^2+^, and growth have also been reported in root hair tips (Monshausen et al., 2007). These findings put forward that one factor (either ROS or wall pH) compensate with at wall dynamics as well as in controlling the cytosolic activities needed to maintain the growth (**Figure 4**).

Lateral root development is controlled by the antagonistic action of auxin and ABA. Auxin promotes the separation of pericycle initials and cell expansion while, ABA is essential during each stage of lateral root growth as it regulates the balance between cell proliferation and differentiation in the root meristem and in lateral root primordia (Dubrovsky et al., 2008; De Rybel et al., 2010; Lavenus et al., 2013). Both hormones also mediate ROS accumulation in growing roots and promote cell expansion. A transcriptomic analysis (Manzano et al., 2014) of roots treated with auxin revealed that majority of the peroxidase genes in the cs-SKP2B (SKP2B is a marker for lateral root development while cs-SKP2B dataset is its specific expression pattern of cell sorting that regulates the developmental program endogenously) dataset are not regulated by ABA. Tsukagoshi et al. (2010) proposed that for growth regulation at root meristem, Prxs activity (regulated by *UPB1*) acts independently of the auxin pathway. In contrast, Ma et al. (2014) advocated that the auxin response involves ROS signaling. Peroxidases take part in lignin formation in the primary cell wall (Marjamaa et al., 2009). It is notable that indole-3 acetic acid (IAA) is easily degraded by plant Prxs (Cosio et al., 2008), which leads to the reduction of auxin pools. In tobacco plant, free IAA levels decreased in roots, indicating that alteration of auxin level is critical for the inhibition of lateral root formation in plants (Moriwaki et al., 2011). Recently, it was proposed that root architecture is determined by the redox-mediated GPX family (Passaia et al., 2014). Moreover, Passaia et al. (2014), through genetic analysis, showed that *gpx* mutants differentially affect lateral root formation (except *gpx3-2* mutants), with all *gpx* mutants showing expanded lateral root primordia (LRP). Passaia et al. (2014) have also observed that *GPX1* and *GPX7* are the two main Prxs to play a significant role in the regulation of lateral root development and root architecture, which is also associated with auxin-dependent control of lateral root formation. In addition, Manzano et al. (2014) have shown that H_2_O_2_ and $O_{2}^{•–}$ actively participate in lateral root development. They have also reported that in the cs-SKP2B dataset, several enzymes are involved in ROS formation, including AtrbohC, cytochrome P450 electron carrier proteins, and lipoxygenases. These findings suggested that ROS signaling is an important part of lateral root development. It can be summarized that Prxs activities (regulated by UPB1) regulate emergence of lateral roots via ROS signaling, most probably by promoting transition from cellular proliferation to differentiation (as discussed in previous section; **Figure 2**).

### ROS and Leaf Growth and Development

The complex coordination between cell proliferation and cell expansion decides the final size of a single organ or organism (Lu et al., 2014). Plant leaves are initiated by the proliferation of meristematic cells and then by cell expansion without additional division in a second phase (Beemster et al., 2005). An equilibrium between negative and positive regulators controls both phases by a large number of genetic pathways. For instance, TFs play a vital role (Townsley and Sinha, 2012). Of course, cell expansion is affected by changes in cell wall architecture and content (Bringmann et al., 2012; Rubio-Diaz et al., 2012). Such alterations may be due to Prxs that alter ROS levels in leaves (Passardi et al., 2004).

The Prxs particularly apoplastic, directly control stiffness of the cell wall, either by promoting or by restricting cellular extension (Tsukagoshi et al., 2010; Lee et al., 2013). In the first scenario, $O_{2}^{•–}$ generated by cell wall peroxidases, promote expansion by cleaving the cell wall polysaccharides and act as cell wall loosening agents (Müller et al., 2009). In contrast, H_2_O_2_ production in the cell wall promotes rigid cross-linking of cell wall components and results in growth restriction or making the cell wall stiffer (Passardi et al., 2004; Lu et al., 2014). The activity and expression of Prxs are suppressed by KUODA1 (KUA1), a MYB-like TF that was found to function as a positive regulator of cell expansion during leaf development by altering apoplastic ROS homeostasis in *A. thaliana* (Lu et al., 2014). The overexpression of KUA1 results in increased cell size with larger leaves without affecting the cell number (Lu et al., 2014). In contrast, the *kua1-1*mutant has smaller leaves than the wild type due to a decrease in cell size, while the cell number was again unaffected. Moreover, the *kua1* mutant shows elevated levels of H_2_O_2_ and increased activity of class III Prxs. The disturbance of KUA1 causes an increase in Prxs activity and results in smaller leaf cells. Therefore, expansion of the cell as well as the final size of the organ is controlled by ROS homeostasis as modulated by KUA1 (Lu et al., 2014). This positive regulation/promotion can be interlinked with change in apoplastic H_2_O_2_ levels and it is noteworthy that changes in H_2_O_2_ level can also affect the $O_{2}^{•–}$ pool (Liszkay et al., 2003). Of note, in the case of KUA1, H_2_O_2_ mediated inhibitory effect of Prxs, seems to be the fundamental link for the regulation of leaf cell expansion. The equilibrium between $O_{2}^{•–}$ and H_2_O_2_ regulates cell proliferation and differentiation zones that decide the size of an organ (as discussed in previous section). Lu et al. (2014) showed that suppression of Prxs expression by KUA1 increased leaf cell expansion, without causing the increase in leaf cell proliferation. Moreover, the size of the first cortical root cell was decreased by H_2_O_2_ treatments (Tsukagoshi et al., 2010), the root-localized Prxs are most probably essential to uphold low H_2_O_2_ levels to support root cell expansion. Contrary to this, Lu et al. (2014) also reported that inhibition of Prxs activity improves leaf size thereby suggesting that apoplastic Prxs mainly produce H_2_O_2_ that leads to cell wall crosslinking in leaves (Passardi et al., 2004). Therefore, the impact of Prxs specifically their effect on the H_2_O_2_ level seems to comprise greater opposing effects on leaf growth in plants.

## Conclusion and Future Directives

In spite of amazing development in our understanding of ROS biology, the exact nature of the ROS-signaling network largely remains mysterious. The present review is an attempt to adress the regulatory action of ROS in plant growth and development. Earlier these were considered to be toxic by-products only, but now they have been found to function as central players in complex signaling networks. We have tried here to uncover the beneficial role of ROS as signaling molecules. Undoubtedly, in the past decade, our understanding in the field of ROS signaling have significantly improved but miles we have to go. We are entering in an exciting era of ROS signaling in plants. Our stage is set and it’s time to fit the different parts of the puzzle into its right place. Definitely, by uncovering novel biological roles of ROS, our understanding in the field of ROS signaling have become more than superficial, but many questions remain to be answered.

• For instance, is it possible to transport ROS from one subcellular site to another, and if possible, how do ROS signals journey within or across different cells?
• How are ROS-modulated gene networks linked with other signaling networks?
• Are they affected by changes in membrane potential?
• How does the effectiveness of ROS signals differ among sub-cellular compartments? How do NADPH oxidases participate in generating these signals?
• What are the concentrations of ROS in different sub-cellular compartments?
• Does each source contribute equally to the cellular ROS pool? Are specific ROS signatures induced by different stimuli?
• How do plant cells sense ROS?
• What are threshold levels of ROS at which they function as signaling molecules?

Answers to these questions are crucial for elucidating mechanisms of ROS signaling. Our current understating regarding the involvement of ROS in developmental processes largely depends on some discovered components of signaling pathways such as NOXs, OXI1, Ca^2+^ influx and eﬄux, and some TFs. Further studies are needed to properly explain the complex regulatory machinery that integrates ROS signals with components of signaling pathways for the regulation of growth and development of plants. ROS are associated with many biotic and abiotic responses, and interpreting ROS signaling is expected to have a noteworthy impact on biotechnology and agriculture, possibly leading to crop development by enhancing yield under suboptimal conditions. Future work will no doubt reveal novel signaling roles for ROS and their interaction with other signals, and hence future of ROS research is very promising.

## Author Contributions

VS and SP have conceptualized the review article. RS, SS, PP, RM, DT, VS, DC, and SP are being involved in writing this review article. RS and SS equally contributed in this review article.

## Conflict of Interest Statement

The authors declare that the research was conducted in the absence of any commercial or financial relationships that could be construed as a potential conflict of interest.
